# Neurocognitive and Psychopathological Predictors of Weight Loss After Bariatric Surgery: A 4-Year Follow-Up Study

**DOI:** 10.3389/fendo.2021.662252

**Published:** 2021-05-05

**Authors:** Emanuela Bianciardi, Giulia Raimondi, Tonia Samela, Marco Innamorati, Lorenzo Maria Contini, Leonardo Procenesi, Mariantonietta Fabbricatore, Claudio Imperatori, Paolo Gentileschi

**Affiliations:** ^1^ Psychiatric Chair, Department of Systems Medicine, University of Rome “Tor Vergata”, Rome, Italy; ^2^ Cognitive and Clinical Psychology Laboratory, Department of Human Science, European University of Rome, Rome, Italy; ^3^ Obesity Unit, Department of Surgery, University of Rome “Tor Vergata”, Rome, Italy

**Keywords:** binge eating disorder, cognitive impairment, executive function, obesity, bariatric surgery, psychopathology, bariatric surgery psychosocial evaluation

## Abstract

Twenty to thirty percent of patients experience weight regain at mid and long-term follow-up. Impaired cognitive functions are prevalent in people suffering from obesity and in those with binge eating disorder, thereby, affecting the weight-loss outcomes. The aim of our study was to investigate neurocognitive and psychopathological predictors of surgical efficacy in terms of percentage of excess weight loss (%EWL) at follow-up intervals of one year and 4-year. Psychosocial evaluation was completed in a sample of 78 bariatric surgery candidates and included psychometric instruments and a cognitive battery of neuropsychological tests. A schedule of 1-year and 4-year follow-ups was implemented. Wisconsin Sorting Card Test total correct responses, scores on the Raven’s Progressive Matrices Test, and age predicted %EWL at, both, early and long-term periods after surgery while the severity of pre-operative binge eating (BED) symptoms were associated with lower %EWL only four years after the operation. Due to the role of pre-operative BED in weight loss maintenance, the affected patients are at risk of suboptimal response requiring ongoing clinical monitoring, and psychological and pharmacological interventions when needed. As a result of our findings and in keeping with the latest guidelines we encourage neuropsychological assessment of bariatric surgery candidates. This data substantiated the rationale of providing rehabilitative interventions tailored to cognitive domains and time specific to the goal of supporting patients in their post-surgical course.

## Introduction

Individuals suffering from obesity reported mild and specific cognitive deficiencies compared to the general population (1, 2). Decreased executive functions, such as problem solving and planning, attention, memory, and inhibitory control were the most cited dysfunctions (3, 4). According to the theory of obesity occurring when the energy homeostasis is “out of balance”, difficulties in these neurocognitive abilities combined with the obesogenic environment may contribute to its pathogenesis (5). In fact, due to the growing abundance of readily available, highly palatable, calorie dense foods, it was advanced that a neurocognitive effort may be required to avoid maladaptive eating behaviors and weight gain (6).

Bariatric surgery is the leading treatment that contributes to the success of battling the obesity epidemic (7, 8). However, in terms of weight loss, the efficacy of surgical interventions may vary significantly between individuals, with 20% to 30% of patients experiencing insufficient weight loss and weight regain at mid and long-term after the operation (9–11). To date, there are only limited studies exploring the role of neurocognition on weight loss after surgery, with the most long-term findings being the association between cognitive dysfunction and suboptimal weight loss at 36-month follow-up (12).

Literature among psychopathological predictors of bariatric surgery efficacy yielded inconsistent results with the exception of some evidence regarding the negative role of binge eating behaviors and depressive symptoms on long-term outcomes (13). In fact, it was highlighted that by the end of the honeymoon period (i.e. the period of rapid and drastic weight loss that begins immediately after surgery and continues for six to 12 months), differences in patients’ weight loss trajectories became apparent (14, 15). More precisely, it was reported that either binge eating disorders (BED) or major depression consistently exhibited their negative effect on body mass index (BMI) evolution at least two years after surgery (16–19).

Yet, these disorders did not represent surgical contraindications since findings were mixed and pathogenic mechanisms were not proven (20). Notably, patients with obesity and BED exhibited impaired executive functioning and inhibitory control compared to those with obesity alone (21). Moreover, psychiatric disorders such as depression were found to affect cognition in bariatric surgery candidates (22).

In view of this, a clear picture of the possible and independent effect of neurocognition, eating disorder, and psychopathology in the long-term period is warranted. Our study aimed to investigate neurocognitive and psychopathological predictors of weight loss after bariatric surgery, both, before and after the honeymoon period.

## Materials and Methods

This research came from a larger prospective study investigating psychiatric aspects of bariatric surgery at the University of Rome, “Tor Vergata”, Italy (23, 24). The present study was performed in accordance with the Helsinki declaration standards and was approved by the Institutional Ethics Review Committee of the University of Rome “Tor Vergata”, with all the participants providing written informed consent.

Our sample consisted of 78 bariatric surgery candidates (59 women and 19 men; mean age: 44.88 ± 11.33 years) referred to the Obesity Unit at the University of Rome “Tor Vergata” for a preoperative psychosocial evaluation. A psychiatrist with training relevant to obesity performed the preoperative psychosocial–behavioral evaluation. Following the national guidelines for bariatric surgery (25), patients with a binge eating disorder and a psychiatric diagnosis were treated at either our center or at their local center. Some patients were prescribed medication, while others, depending on the severity of their symptoms, were submitted to psychotherapy. The clearance to surgery is made of a written report which is given to the patient at the end of the evaluation/treatment pathway (26). All patients were administered psychometric instruments and a cognitive battery of neuropsychological tests. Postoperatively, patients attended follow-ups with the surgical staff at 12 (mean; min-max: 13.8; 12-24) and 48-month (mean; min-max: 55.2; 48-71) periods after surgery. At the second follow-up period, 17 participants were lost (total sample=61).

Preoperatively and at the two follow-up visits the surgical staff calculated body mass index (BMI) and the percentage of excess weight loss (%EWL) measuring participants’ height and weight in their street clothes.

### Psychometric Instruments

The Italian version of the Symptom Checklist 90-Revised (SCL-90-R) was used (27). SCL-90-R is a 90-item self-report on a 5-point Likert scale (0-4) assessing general psychopathology and emotional distress that has been widely used in bariatric surgery candidates (28, 29). It provides a global severity index (GSI-90) proposed as an index of overall psychological distress, with higher scores reflecting higher levels of psychopathological distress as well as greater severity of self-reported symptoms. Cronbach’s alpha in the present sample was 0.97.

The Italian version of the Binge Eating Scale (BES) was administered (30). The BES is a 16-item self-report questionnaire assessing the severity of binge eating behavior. Total scores range from 0 to 46 with cut-off scores of possible (18- 26) and probable (≥ 27) BED (31). Cronbach’s alpha in the present sample was 0.91.

### Neuropsychological Tests

The computerized version of the Wisconsin Sorting Card Test (WCST) was used to measure executive domains (32). Four stimulus cards with different symbols (in terms of color, number, and shape) are displayed on the computer screen. Participants are requested to match a response card with one of the four stimulus cards according to a criterion (which can be color, number, or form). After 10 consecutive correct pairings, the criterion for matching the cards changed. The task continues until all 128 response cards have been displayed. Different indices can be calculated: “total categories completed’’, ‘‘perseverative errors’’, ‘‘total non-perseverative errors’’, ‘‘total correct responses’’, ‘‘number of perseverative responses’’, and ‘‘the total number of errors’’.

The Raven’s Progressive Matrices Test (SPM) is one of the most widely used instruments measuring general intelligence (33) and was also adopted in the pre-surgical psychosocial evaluation (34). SPM is relatively independent of education or cultural influence. It takes almost 40 minutes to complete. The task consists of several visual analogy problems. Each item consists of a matrix of geometric designs representing the problem with one design removed from the sequence. Participants are invited to glean the kind of relationship between designs and choose the missing figure from among the alternative set. SPM includes five sets (A through E) progressing in difficulty.

The Continuous Performance Test II (CPT) (35) is a computer-administered task that measures sustained attention and impulsivity. The subject is asked to respond every time a specific letter appears on the screen (target stimulus) and not to respond when different stimuli appear on the screen (distractors). Errors can be rated as responses to non-target stimuli (commission errors) and as failure to respond to target stimuli (omission errors). Six scores are computed: omission errors, commission errors (response to non-target stimulus), reaction times (average response time in milliseconds to stimuli), standard deviation in reaction time indicating variability of the performance, and warning (ability to discriminate between target and non-target).

The verbal fluency test includes two tasks (namely semantic and phonemic fluency tasks) (36): Letter (LF) (37) and Category (CF) verbal Fluency (38) which were used to assess both verbal ability and executive control. LF consists of producing as many words as possible in response to a specific letter. CF consists of producing as many words as possible in response to a specific semantic category. These tasks permit an evaluation of the ability to generate words and investigate the ability of the responder to form a strategy in response to a required task. Verbal fluency scores are computed as the number of correct words produced in 1 minute.

Digit Span (39) was used to assess the short-term memory ability. The examiner reads a sequence of numbers to the participants, who are required to repeat the same sequence. If subjects correctly repeat the sequence of numbers, the examiner moves on to the next sequence increasing the span of numbers that the participants have to repeat.

Rey Auditory Verbal Learning Test (RAVLT) (40) assessed patients’ verbal memory. The RAVLT is widely used to evaluate the nature and severity of memory dysfunction. The task is divided into two sections. In the first section, the examiner reads a list of 15 words to the participant who has to immediately recall as many words as possible. This procedure is repeated five times. In the second section, the examiner reads the list, this time there is a delay of 15 minutes before the participant is requested to recall the words. The test gives two quantitative measures: the immediate recall (Rey-T0) and delayed recall of the list (Rey-T15).

The Trail Making Test (TMT) was performed to assess visual attention, mental flexibility, and executive functioning (41). It consists of two parts (A-B). Part A is a good measure of rote memory. Part B is more sensitive to executive functioning.

The Raven’s Progressive Matrices Test was performed in one session, with other tests being performed in a second session.

### Statistical Analysis

All the analyses were performed with the Statistical Package for the Social Sciences (SPSS) 25.0 (IBM, Armonk, NY, USA). Relationships between variables were computed through Pearson’s indices of associations (*r*). Pearson’s *r* coefficients < 0.10 are considered a negligible or null effect, *r* between 0.10 and 0.30 a small effect, *r* between 0.30 and 0.50 a medium effect, and *r* ≥ 0.50 a large effect (42). We conducted both zero order and first order correlations, controlling for sex, given that 75.6% of the total sample was composed of women (43). Hierarchical linear regression analyses were performed to investigate whether clinical and cognitive variables were significant predictors of percentage of excess weight loss at the first (%EWL I) and second (%EWL II) follow up, while controlling sociodemographic variables (i.e. age, sex, school attainment). The independent variables were entered into the regression model according to the following blocks: (1) cognitive variables; and (2) sociodemographic and clinical variables. The associations were reported as standardized beta coefficients (β) and their *p*-values.

Since different surgery procedures may lead to different weight loss outcomes (44) and 82% of our total sample (n=64) being composed of patients undergoing sleeve gastrectomy, we conducted additional analyses on those participants undergoing this surgery. Among the subsample of sleeve gastrectomy patients, we conducted both zero order and first order correlations, controlling for sex. Hierarchical linear regression analyses were performed to investigate whether sociodemographic (i.e. age, sex, school attainment), clinical and cognitive variables were significant predictors of a percentage of excess weight loss at, both, the first (%EWL I) and second (%EWL II) follow up.

According to the last observation carried forward (LOCF) approach, missing values for % EWL at the second follow-up were replaced with the last available observation (45).

## Results

### Characteristics of the Sample

Average BMI of the patients undergoing bariatric surgery was 43.15 Kg/m^2^ (SD = 6.01; Range= 33.26-55.83). According to the BMI cut-off of the International Classification of Diseases (ICD) 8 (10.3%) patients were included in class I obesity (i.e., 30 Kg/m^2^ ≤ BMI ≤ 34.99 Kg/m^2^), 20 (25.6%) patients in class II obesity (i.e., 35 Kg/m^2^ ≤ BMI ≤ 39.99 Kg/m^2^), and 50 (64.1%) in class III obesity (i.e., BMI ≥ 40 Kg/m^2^). Eleven patients (14.3%) met the criteria for moderate levels of binge eating (18 ≤ BES ≤ 26), and 8 (10.1%) had severe levels of binge eating (BES ≥ 27) (see **Table 1**).

**Table 1:** Descriptive statistics of the sample (N=78).

Variables	Count/M	%/(SD)
**Age** M(SD)	44.85	(11.23)
**Sex** N/%		
Men	19	24.6
Women	59	75.6
**School attainment ≥ 13 years** N/%	35	44.9
**BMI pre-surgery** M(SD)	43.15	(6.01)
Obesity class I (30 Kg/m^2^ ≤ BMI ≤ 34.99 Kg/m^2^) N/%	8	10.3
Obesity class II (35 Kg/m^2^ ≤ BMI ≤ 39.99 Kg/m^2^) N/%	20	25.6
Obesity class III (BMI ≥ 40 Kg/m^2^) N/%	50	64.1
**%EWL I** M (SD)	58.13	(27.16)
**%EWL II** M (SD)	54.83	(27.11)
**BES** M (SD)	13.09	(10.62)
BES ≤ 17 N/%	59	75.6
18 ≤ BES ≤ 26 N/%	11	14.3
BES ≥ 27 N/%	8	10.1
**SCL-GSI** M (SD)	1.12	(0.10)

M, Mean; SD, Standard Deviation; %, Percentage; BMI, Body Mass Index; %EWL I, percentage of excess weight loss at the first follow-up; %EWL II, percentage of excess weight loss at the second follow-up; BES, Binge Eating Scale; SCL GSI, Symptom Checklist-90 Revised - Global Severity Index.

Descriptive statistics for the subsample of patients undergoing sleeve gastrectomy procedures were included in the Supplementary material (see **Table S1**). Average BMI of the patients undergoing sleeve gastrectomy procedures was 43 Kg/m^2^ (SD = 5.83; Range= 33.26-55.83). According to the BMI cut-off of the International Classification of Diseases (ICD) 4 (6.3%) patients were included in class I obesity (i.e., 30 Kg/m^2^ ≤ BMI ≤ 34.99 Kg/m^2^), 17 (26.5%) patients in class II obesity (i.e., 35 Kg/m^2^ ≤ BMI ≤ 39.99 Kg/m^2^), and 43 (67.2%) in class III obesity (i.e., BMI ≥ 40 Kg/m^2^). Eight patients (12.5%) met the criteria for moderate levels of binge eating (18 ≤ BES ≤ 26), and 5 (7.8%) had severe levels of binge eating (BES ≥ 27).

### Variables Associated With the Percentage of Excess Weight Loss (%EWL) at 1-Year and 4-Year Follow-Up

Correlations among %EWL I, %EWL II and sociodemographic and clinical variables are reported in **Table 2**, and correlations among %EWL I, %EWL II and cognitive variables are reported in **Table 3**. Both the %EWL I (*r* = -0.27, *p* < 0.05) and %EWL II (*r* = -0.31, *p* < 0.01) were negatively and significantly correlated with age. The associations with BES scores and the SCL GSI were nonsignificant for both %EWL I and %EWL II. In partial correlations, when controlling for sex, both the %EWL I (*r* = -0.26, *p* < 0.05) and %EWL II (*r* = -0.32, *p* < 0.01) were still negatively and significantly correlated with age, and the associations with BES scores and the SCL GSI for both %EWL I and %EWL II were still nonsignificant.

**Table 2:** Associations between %EWL at I and II follow-up, socio-demographic and clinical variables (N=78).

	%EWL I	%EWL II
**Age**	-0.27*(-0.26*)	-0.31**(-0.32**)
**School attainment**	-0.03 (-0.03)	-0.03 (-0.03)
**Sex**	-0.08	-0.12
**BMI pre-surgery**	0.13 (0.12)	0.18 (0.17)
**BES**	0.09 (-0.08)	-0.20 (-0.20)
**SCL-GSI**	0.08 (0.05)	0.09 (0.03)

*p<0.05; **p<0.001; Bold values indicate significant variable.

Between parentheses partial correlation values when controlling for sex.

%EWL I, percentage of excess weight loss at the first follow-up; %EWL II, percentage of excess weight loss at the second follow-up; BMI, Body Mass Index; BES, Binge Eating Scale; SCL-GSI, Symptom Checklist-90 Revised - Global Severity Index.

**Table 3:** Associations between %EWL at I and II follow-up and cognitive variables (N=78).

	%EWL I	%EWL II
**Rey-T0**	-0.05 (-0.09)	-0.05 (-0.12)
**Rey-T15**	0.05 (0.04)	0.05 (0.02)
**Phonemic VF**	0.18 (0.17)	0.09 (0.07)
**Semantic VF**	0.07 (0.08)	0.01 (-0.01)
**TMT-A**	0.11 (0.09)	0.19 (0.18)
**TMT-B**	0.02 (0.00)	0.02 (-0.01)
**TMT B-A**	0.03 (-0.00)	0.01 (-0.02)
**DS**	0.07 (0.13)	0.11 (0.10)
**SPM-S**	0.10 (0.13)	0.11 (0.14)
**SPM-E**	0.22* (0.24*)	0.22 (0.25*)
**CPT Omission**	-0.06 (-0.05)	-0.04 (-0.03)
**CPT Commission**	0.04 (0.08)	-0.03 (0.00)
**CPT Hit**	-0.15 (-0.15)	-0.15 (-0.14)
**CPT-SD**	-0.23* (-0.21)	-0.22 (-0.21)
**CPT Perseveration**	-0.11 (-0.09)	-0.10 (-0.08)
**CPT Detectability**	0.18 (0.06)	-0.07 (-0.02)
**WCST**	0.28* (0.26*)	0.21 (0.15)
**WCST Er**	0.09 (0.06)	0.09 (0.02)
**WCST Perseveration**	0.13 (0.09)	0.09 (0.02)
**WCST-P Er**	0.11 (0.08)	0.09 (0.02)
**WCST Non-P Er**	0.10 (0.07)	0.11 (0.05)
**WCST-cc**	0.14 (0.12)	0.10 (0.04)

*p<0.05; Bold values indicate significant variables.

Between parentheses partial correlation values when controlling for sex.

%EWL I, percentage of excess weight loss at the first follow up; %EWL II, percentage of excess weight loss at the second follow up; Rey-T0, Rey Auditory Verbal Learning Test at time 0; Rey-T15, Rey Auditory Verbal Learning Test after 15 minutes; Phonemic VF, Phonological Verbal Fluency; Semantic VF, Semantic Verbal Fluency; TMT-A, Trail Making Test part A; TMT-B, Trail Making Test part B; TMT B-A, Trail Making Test time ratio between part B and A; DS, Digit Span; SPM-S, Raven’s Standard Progressive Matrices education corrected; SPM-E, Raven’s Standard Progressive Matrices age corrected; CPT, Continuous Performance Test; CPT Hit, Continuous Performance Test Reaction Time; CPT-SD, Continuous Performance Test Standard Error; CPT Perseveration, Continuous Performance Test Perseverative responses; WCST, Wisconsin Card Sorting Test total score; WCST Er, Wisconsin Card Sorting Test total error; WCST Perseveration, Wisconsin Card Sorting Test Perseverative responses; WCST-P Er, Wisconsin Card Sorting Test Perseverative Error responses; WCST Non-P Er, Wisconsin Card Sorting Test Non Perseverative Error responses; WCST-cc; Wisconsin Card Sorting Test Correct Categories completed.

The %EWL I correlated significantly and positively with SPM-E scores (*r* = 0.22, *p* < 0.05) and WCST total correct responses (*r* = 0.28, *p* < 0.05), and negatively with the CTP-SD (*r* = -0.23, *p* < 0.05). When controlling for sex, %EWL I remained significantly and positively correlated with SPM-E scores (*r* = 0.24, *p* < 0.05) and WCST total correct responses (*r* = 0.26, *p* < 0.05), but the correlation with the CTP-SD (*r* = -0.21, *p* = 0.06) became nonsignificant. Furthermore, at the zero-order correlations, %EWL II did not correlate with any of the cognitive variables, and only when controlling for sex, %EWL II significantly and positively correlated with SPM-E scores (*r* = 0.25, *p* < 0.05). However, all these correlations were relatively weak (*r* < 0.40).

Additional analyses were performed on patients who underwent sleeve gastrectomy procedures (reported in **Tables S2 **and** S3**). %EWL II was negatively and significantly associated only with age (*r* = -0.29, *p* < 0.05). All the remaining associations with sociodemographic and clinical variables were nonsignificant. %EWL II (*r* = -0.26, *p* < 0.05) was still negatively and significantly associated with age even when controlling for sex. %EWL I was associated significantly and positively with SPM-E scores (*r* = 0.25, *p* < 0.05) and WCST total correct responses (*r* = 0.32, *p* < 0.01), even when controlling for sex (SPM-E: *r* = 0.29, *p* < 0.05; WCST total correct responses *r* = 0.34, *p* < 0.01).

### Predictors of the Percentage of Excess Weight Loss at I Follow-Up

Significant variables at the bivariate analyses were included as predictors in a hierarchical linear regression model, with %EWL I as the criterion. Although nonsignificant at the bivariate analyses, we also included in the regression model, sex, school attainment, and BMI pre-surgery. The model fitted the data well (Block 1: *F* = 4.186; *p* < 0.01. Block 2: *F* = 3.652; *p* < 0.01; see **Table 4**) and explained between 11% and 23% of the variance of the data (Block 1: *R* ^2^ *Change* = 0.15; *p* < 0.01. Block 2: *R* ^2^ *Change* = 0.16; *p* < 0.05). WCST total correct responses (*β* = 0.31; *p =* 0.007) were independently associated with %EWL I even when controlling for the presence of sociodemographic and clinical variables. SPM-E (*β* = 0.33; *p =* 0.004) was independently associated with %EWL I only when controlling for the presence of sociodemographic and clinical variables. Among the other variables included in the model, only age (*β* = -0.32; *p* = 0.01) was independently associated with %EWL I.

**Table 4:** Hierarchical linear regression analysis predicting the percentage of excess weight loss at the first follow-up (N=78).

Dependent Variable: %EWL I	*β*	*p*	[95% CI]	Adjusted *R^2^*	*F*	Significance	*R^2^* Change	*F* Change	Significance
**Block 1 independent variables**				0.11	Df_3;69_ = 4.186	p < 0.01	0.15	4.186	p < 0.01
**SPM-E**	0.23	0.05	[-0.001; 0.947]						
**CPT-SD**	-0.09	0.44	[-0.528; 0.234]						
**WCST**	**0.26**	**0.02**	[0.075; 1.248]						
**Block 2 independent variables**				0.23	Df_8;64_ = 3.652	p < 0.01	0.16	2.972	p < 0.05
**SPM-E**	**0.33**	**0.004**	[0.225; 1.141]						
**CPT-SD**	0.005	0.96	[-0.392; 0.408]						
**WCST**	**0.31**	**0.007**	[0.229; 1.378]						
**Age**	**-0.32**	**0.01**	[-1.397; -0.175]						
**Sex**	0.05	0.60	[-10.370; 17.648]						
**School attainment**	-0.19	0.09	[-3.386; 0.284]						
**BMI pre-surgery**	0.17	0.12	[-0.220; 1.765]						
**BES**	-0.21	0.06	[-1.132; 0.022]						

Bold values indicate significant variable. %EWL I, percentage of excess weight loss at the first follow-up; SPM-E, Raven’s Standard Progressive Matrices age corrected; CPT-SD, Continuous Performance Test Standard Error; WCST, Wisconsin Card Sorting Test total score; BMI, Body Mass Index; BES, Binge Eating Scale.

Additional analyses were performed separately on patients undergoing sleeve gastrectomy procedures (reported in **Table S4**). The multivariate model fitted the data well (Block 1: *F* = 7.949; *p* < 0.01. Block 2: *F* = 2.454; *p* < 0.05). However, the inclusion of sociodemographic variables did not increase the variance explained by the model (*F Change* = 0.553, p = 0.73). Conversely, the variance explained decreased nonsignificantly from 17% to 14%. This result could indicate a specification error in the model related to one or more of the sociodemographic variables included. In fact, the standard error (SE) associated with the variation in sex was 6.04. WCST total correct responses (*β* = 0.34; *p =* 0.01) and SPM-E (*β* = 0.31; *p =* 0.02) were independently associated with %EWL I even when controlling for the presence of sociodemographic and clinical variables.

### Predictors of the Percentage of Excess Weight Loss at II Follow-Up

Although only age was significant at the bivariate analyses, other sociodemographic, clinical and cognitive variables were included as predictors in a hierarchical linear regression model, with %EWL II as criterion. The model fitted the data well (Block 1: *F* = 2.925; *p* < 0.05. Block 2: *F* = 4.866; *p* < 0.001) and explained between 7% and 30% of the variance of the data (Block 1: *R* ^2^ *Change* = 0.11; *p* < 0.05. Block 2: *R* ^2^ *Change* = 0.26; *p* < 0.001. See **Table 5**). In the first block none of the cognitive variables were significantly associated with %EWL II. In the second block, when controlling for socio-demographic and clinical variables, SPM-E scores (*β* = 0.36; p = 0.001) and WCST total correct responses (*β* = 0.23; *p* = 0.03) were independently associated with %EWL II. Among other variables included in the model, age (*β* = -0.41; *p* = 0.001) and BES (*β* = -0.30; *p* = 0.006) were independently associated with %EWL II.

**Table 5:** Hierarchical linear regression analysis predicting the percentage of excess weight loss at the second follow up (N=78).

Dependent Variable: %EWL II	*β*	*p*	[95% CI]	Adjusted *R^2^*	*F*	Significance	*R^2^* Change	*F* Change	Significance
**Block 1 independent variables**				0.07	Df_3;69_ = 2.925	p < 0.05	0.11	2.925	p < 0.05
**SPM-E**	0.22	0.06	[-0.024; 0.922]						
**CTP-SD**	-0.12	0.32	[-0.569; 0.191]						
**WCST**	0.15	0.20	[-0.211; 0.959]						
**Block 2 independent variables**				0.30	Df_8;64_ = 4.866	p < 0.001	0.26	5.463	p < 0.001
**SPM-E**	**0.36**	**0.001**	[0.294; 1.154]						
**CTP-SD**	0.02	0.86	[-0.342; 0.406]						
**WCST**	**0.23**	**0.03**	[0.033; 1.122]						
**Age**	**-0.41**	**0.001**	[-1.550; -0.417]						
**Sex**	0.02	0.80	[-11.402; 14.571]						
**School attainment**	-0.21	0.05	[-3.356; 0.046]						
**BMI pre-surgery**	0.20	0.05	[-0.034; 1.806]						
**BES**	**-0.30**	**0.006**	[-1.288; -0.219]						

Bold values indicate significant variable. %EWL II, percentage of excess weight loss at the first follow up; SPM-E, Raven’s Standard Progressive Matrices age corrected; CPT-SD, Continuous Performance Test Standard Error; WCST, Wisconsin Card Sorting Test total score; BMI, Body Mass Index; BES, Binge Eating Scale.

The same analysis was performed on patients undergoing sleeve gastrectomy procedures (reported in **Table S5**). Model 2 fitted the data well (Block 2: *F* = 2.195; *p* < 0.05) while model 1 did not (Block 1: *F* = 3.062; *p* = 0.054). However, the increase in the variance explained by model 2 (*R* ^2^ *Change* = 0.12, *F Change* = 1767, p = 0.13) was nonsignificant when compared to model 1. As reported for the previous model with %EWL I as the dependent variable, the standard error associated with gender was high (SE = 6.71). WCST total correct responses (*β* = 0.25; *p =* 0.04) was the only variable independently associated with %EWL II.

## Discussion

We documented that cognitive impairment predicted weight-loss at the early (i.e., one year) and long-term (i.e., >four years) periods after surgery, while the severity of pre-operative binge eating behavior did not emerge as a risk factor for decrease in weight-loss until four years after the operation. To the best of our knowledge, among studies investigating the neurocognitive predictors of bariatric surgery, at 55 months, we have provided the longest follow-up. Apart from binge eating symptoms, general psychopathology was not correlated with weight-loss outcomes. Accordingly, bariatric surgery is a viable treatment for those suffering from a psychiatric disorder in terms of weight-loss outcomes. Despite this, it is recommended to have a close and ongoing monitoring of these vulnerable patients, due to the increased risk of depression and self-harm after surgery (46). Moreover, we reinforced the observation that bariatric surgery is less effective for older patients (47), which was previously attributed to the longer duration of comorbidities and impaired metabolic functioning compared to young people (48, 49). Our findings differed from and added to past results which reported an association between cognitive impairment and poorer bariatric surgery outcomes.

We highlighted various strengths of this study. First of all, participants underwent an exhaustive battery of cognitive tests assessing different areas of cognitive function: mental flexibility, working memory, attention, concentration, verbal learning and memory, verbal fluency, impulse control as well as intellectual capacity which were separately investigated in previous studies (50, 51).

In exploring the possible influence of all the above cognitive domains, we were able to achieve the significant prediction of executive functions and basic cognitive level as measured by WCST and SPM tests on short and long-term weight loss. Executive functions mainly carried out by the prefrontal cortex can be defined as “high level” activities that modulate “lower-level” subcortical responses (52). Executive functions such as decision making, response inhibition, and cognitive flexibility, consist of mental capacities necessary to engage in the planning for and achieving of goal-directed activities. If executive functions are intact, people can have the capacity to complete a life-plan and be productive. On the other hand, the impairment or loss of these functions restricts the ability to maintain a proper self-improving, and productive life (53). Although we did not provide data among post-operative eating habits of patients (54), it was proven that brain circuits involved in executive control might be a distinctive determinant of successful surgical outcome (55). Accordingly, since executive domains are pivotal to the adjustment in novel situations, the anatomical change which was imposed by surgery may force a new eating regimen representing an impenetrable target for these patients. The lack of patients adaptation to the post-surgery dietary and behavioral recommendations may result in suboptimal outcomes in terms of both weight loss and surgical complications (56–58).

Moreover, compared to expedient studies on cognitive impairment, the second merit of our research was the longer follow-up time and the two-step assessment. This strategy allowed us to go largely beyond and to distinguish the *ante* from the *post*-honeymoon period which was typically fixed around 12 to 18 months after surgery (59). This period can be selected as the first “critical” window during which weight regain might appear (60).

Another strong point of our study was the contrast between the cognitive and the psychopathological impairment effect on weight loss trajectory. The following results deepen the insight on how and when different clusters of predictors may exhibit their influence on weight loss outcomes. Then, a long time after surgery, old eating attitudes, such as binge behaviors, may generate their harmful effect. Whereas cognitive impairment was found to be relevant to the nature of BED (61, 62), differentiating their effects also reduced possible confounding issues.

Beyond the worth of our study, we must recognize its limitations. Due to the high prevalence (82% of the total sample) of sleeve gastrectomy procedures, we were unable to analyze the effect of potential interaction between type of operation and cognitive deficits on the evolution of BMI over the two follow-up periods (57, 63, 64). Moreover, we did not perform the cognitive battery test after surgery, neglecting the possibility of measuring changes in cognitive functioning associated with weight-loss (65). Furthermore, the sample was quite small when considering the number of neurocognitive and clinical variables investigated. Finally, data regarding patients’ adherence to nutritional follow-ups were not available.

Nonetheless, as a result of the above findings and in keeping with the latest guideline (66), we should encourage neuropsychological assessment of bariatric surgery candidates and, consequently, appropriate treatments, such as cognitive remediation therapy, should be arranged post-operatively. Cognition demonstrated a strong and bidirectional relationship with obesity (67), thus, its role in the bariatric surgery field may substantiate the rationale of providing rehabilitative interventions. Such treatments might be tailored to cognitive domains and time specific to the goal of supporting patients in their post-surgical course (68).

## Conclusion

In conclusion, our findings answered the need to analyze the interactions among all factors involved in predicting pathways of suboptimal weight loss after surgery, such as demographics, psychopathology, eating disorders, and cognitive functioning. Additionally, we limited the possible overlap between cognitive impairment and eating disorders which were independently and at different times associated with poorer weight loss outcomes.

Neither psychopathology nor BED should prevent surgery; however, due to the crucial implications of BED for the prognosis, it is worth noting that this disorder is manageable through psychological and pharmacological interventions. Cognitive functioning needs to be addressed preoperatively and may be improved by appropriate rehabilitative techniques.

Finally, with the two-step follow-up, our findings provided the challenge to better understand how cognitive and psychopathological domains of patients might interact with weight loss and weight loss maintenance.

## Data Availability Statement

The raw data supporting the conclusions of this article will be made available by the authors, without undue reservation.

## Ethics Statement

The studies involving human participants were reviewed and approved by University of Rome Tor Vergata. The patients/participants provided their written informed consent to participate in this study.

## Author Contributions

EB and PG designed the study, wrote the manuscript, and performed psychiatric and surgical assessments. GR and TS performed the statistics, wrote the results, and contributed to the interpretation of data and to the discussion section. MI supervised statistical analyses and contributed to the first draft of the paper. LP and LC collected psychiatric and cognitive tests and completed the database. MI, MF, and CI revised the manuscript. All authors contributed to the article and approved the submitted version.

## Conflict of Interest

The authors declare that the research was conducted in the absence of any commercial or financial relationships that could be construed as a potential conflict of interest.
