# Supplementary material for: Neurocognitive and Psychopathological Predictors of Weight Loss After Bariatric Surgery: A 4-Year Follow-Up Study
Source: Front Endocrinol (Lausanne). 2021 May 5;12:662252. doi: 10.3389/fendo.2021.662252 (PMC8131828; doi:10.3389/fendo.2021.662252)
Supplement: Supplementary file 1 [file DataSheet_1.docx]

**Table S1.** Descriptive statistics of the patients undergoing sleeve gastrectomy procedures (N=64).

| **Variables** | **Count/M** | **%/(SD)** |
| --- | --- | --- |
| **Age** M(SD) | 44.11 | (11.00) |
| **Sex** N/%  Man  Women | 18  46 | 28.1  71.9 |
| **School attainment ≥ 13 years** N/% | 35 | 44.9 |
| **BMI pre-surgery** M(SD)  Obese class I (30 Kg/m^2^ ≤ BMI ≤ 34.99 Kg/m^2^) N/%  Obese class II (35 Kg/m^2^ ≤ BMI ≤ 39.99 Kg/m^2^) N/%  Morbid obese class III (BMI ≥ 40 Kg/m^2^) N/% | 43.49  4  17  43 | (5.83)  6.3  26.5  67.2 |
| **%EWL I** M(SD) | 63.47 | (21.51) |
| **%EWL II** M(SD) | 59.08 | (23.67) |
| **BES** M(SD)  BES ≤ 17 N/%  18 ≤ BES ≤ 26 N/%  BES ≥ 27 N/% | 12.31  47  8  5 | (9.92)  79.7  12.5  7.8 |
| **SCL-GSI** M(SD) | 1.13 | (0.11) |
| M, Mean; SD, Standard Deviation; %, Percentage; BMI, Body Mass Index; %EWL I, percentage of weight loss at the first follow up; %EWL II, percentage of weight loss at the second follow up; BES, Binge Eating Scale; SCL GSI, Symptom Checklist-90 Revised - Global Severity Index. | | |

**Table S2.** Associations between %EWL at I and II follow up and socio-demographic and clinical variables (N=64)

|  | **%EWL I** | **%EWL II** |
| --- | --- | --- |
| **Age** | -0.21 (-0.22) | **-0.29* (-0.26*)** |
| **School attainment** | -0.04 (0.08) | -0.03 (-0.01) |
| **Gender** | 0.03 | -0.04 |
| **BMI pre-surgery** | -0.13 (-0.8) | 0.04 (0.03) |
| **BES** | 0.18 (0.24) | -0.003 (-0.05) |
| **SCL-GSI** | 0.02 (-0.03) | 0.10 (-0.05) |
| *p<0.05; Bold values indicate significant variable.  Between parentheses partial correlations values when controlling for sex.  %EWL I, percentage of weight loss at the first follow up; %EWL II, percentage of weight loss at the second follow up; BMI, Body Mass Index; BES, Binge Eating Scale; SCL-GSI, Symptom Checklist-90 Revised - Global Severity Index. | | |

**Table S3.** Associations between %EWL at I and II follow up and cognitive variables (N=64).

|  | **%EWL I** | **%EWL II** |
| --- | --- | --- |
| **Rey-T0** | -0.03 (-0.10) | -0.08 (-0.20) |
| **Rey-T15** | 0.01 (-0.09) | -0.06 (-0.18) |
| **Phonological VF** | 0.12 (0.08) | 0.04 (-0.006) |
| **Semantic VF** | -0.02 (-0.03) | -0.01 (-0.03) |
| **TMT-A** | 0.04 (0.009) | 0.18 (0.18) |
| **TMT-B** | -0.04 (-0.06) | -0.03 (-0.07) |
| **TMT Diff** | -0.02 (-0.04) | -0.07 (-0.12) |
| **DS** | 0.05 (0.14) | 0.12 (0.10) |
| **SPM-S** | 0.20 (0.23) | 0.10 (0.14) |
| **SPM-E** | **0.25* (0.29*)** | 0.16 (0.19) |
| **CPT Omission** | -0.16 (-0.16) | -0.06 (-0.04) |
| **CPT Commission** | 0.10 (-0.07) | -0.14 (-0.10) |
| **CPT Hit** | -0.10 (-0.11) | -0.11 (-0.11) |
| **CPT-SD** | -0.24 (-0.23) | -0.18 (-0.18) |
| **CPT Perseveration** | -0.19 (-0.18) | -0.13 (-0.11) |
| **CPT Dectability** | 0.10 (-0.08) | -0.17 (-0.13) |
| **WCST** | **0.32** (0.34**)** | 0.24 (0.20) |
| **WCST Er** | 0.11 (0.08) | 0.06 (-0.02) |
| **WCST Perseveration** | 0.09 (0.04) | 0.02 (-0.07) |
| **WCST-P Er** | 0.08 (0.04) | 0.02 (-0.07) |
| **WCST Non-P Er** | 0.17 (0.15) | 0.12 (0.06) |
| **WCST-cc** | 0.21 (0.20) | 0.09 (0.02) |
| *p<0.05; **p<0.01; Bold values indicate significant variables.  Between parentheses partial correlations values when controlling for sex.  %EWL I, percentage of weight loss at the first follow up; %EWL II, percentage of weight loss at the second follow up; Rey-T0, Rey Auditory Verbal Learning Test at time 0; Rey-T15, Rey Auditory Verbal Learning Test after 15 minutes; Phonemic VF, Phonological Verbal Fluency; Semantic VF, Semantic Verbal Fluency; TMT-A, Trail Making Test part A; TMT-B, Trail Making Test part B; TMT Diff, Trail Making Test time difference between part B and A; DS, Digit Span; SPM-S, Raven’s Standard Progressive Matrices education corrected; SPM-E, Raven’s Standard Progressive Matrices age corrected; CPT, Continuous Performance Test; CPT Hit, Continuous Performance Test Reaction Time; CPT-SD, Continuous Performance Test Standard Error; CPT Perseveration, Continuous Performance Test Perseverative responses; WCST, Winsconsin Card Sorting Test total score; WCST Er, Winsconsin Card Sorting Test total error; WCST Perseveration, Winsconsin Card Sorting Test Perseverative responses; WCST-P Er, Winsconsin Card Sorting Test Perseverative Error responses; WCST Non-P Er, Winsconsin Card Sorting Test Non Perseverative Error responses; WCST-cc; Winsconsin Card Sorting Test Correct Categories completed. | | |

**Table S4.** Hierarchical linear regression analysis predicting the percentage of weight loss at the first follow up (N=64).

| Dependent Variable: %EWL I | | | | | | | | | |
| --- | --- | --- | --- | --- | --- | --- | --- | --- | --- |
|  | ***β*** | ***p*** | **[95% CI]** | **Adjusted *R^2^*** | ***Test*** | **Significance** | ***R^2^* Change** | ***F* Change** | **Significance** |
| Block 1 independent variables |  |  |  | 0.17 | F_2;58_ = 7.494 | p < 0.01 | 0.20 | 7.494 | p < 0.01 |
| SPM-E | **0.28** | **0.01** | [0.090; 0.883] |  |  |  |  |  |  |
| WCST | **0.35** | **0.003** | [0.241; 1.160] |  |  |  |  |  |  |
| Block 2 independent variables |  |  |  | 0.14 | F_7;53_ = 2.454 | p < 0.05 | 0.03 | 0.425 | p > 0.73 |
| SPM-E | **0.31** | **0.02** | [0.082; 0.966] |  |  |  |  |  |  |
| WCST | **0.34** | **0.01** | [0.145; 1.192] |  |  |  |  |  |  |
| Age | -0.18 | 0.17 | [-0.873; 0.164] |  |  |  |  |  |  |
| Sex | 0.13 | 0.30 | [-5.883; 18.342] |  |  |  |  |  |  |
| School attainment | -0.05 | 0.69 | [-2.128; 1.418] |  |  |  |  |  |  |
| BMI pre-surgery | 0.05 | 0.65 | [-0.722; 1.136] |  |  |  |  |  |  |
| BES | -0.03 | 0.79 | [-0.496; 0.643] |  |  |  |  |  |  |
| Bold values indicate significant variable. %EWL I, percentage of weight loss at the first follow up; SPM-E, Raven’s Standard Progressive Matrices age corrected; WCST, Winsconsin Card Sorting Test total score; BMI, Body Mass Index; BES, Binge Eating Scale. | | | | | | | | | |

**Table S5.** Hierarchical linear regression analysis predicting the percentage of weight loss at the second follow up (N=64).

| Dependent Variable: %EWL II | | | | | | | | | |
| --- | --- | --- | --- | --- | --- | --- | --- | --- | --- |
|  | ***β*** | ***p*** | **[95% CI]** | **Adjusted *R^2^*** | ***Test*** | **Significance** | ***R^2^* Change** | ***F* Change** | **Significance** |
| Block 1 independent variables |  |  |  | 0.06 | F_2;58_ = 3.062 | P = 0.054 | 0.09 | 3.062 | P = 0.054 |
| SPM-E | 0.18 | 0.14 | [-0.117; 0.811] |  |  |  |  |  |  |
| WCST | **0.25** | **0.049** | [0.001; 1.076] |  |  |  |  |  |  |
| Block 2 independent variables |  |  |  | 0.12 | F_7;53_ = 2.195 | P < 0.05 | 0.13 | 1.767 | P = 0.13 |
| SPM-E | **0.29** | **0.03** | [0.052; 1.018] |  |  |  |  |  |  |
| WCST | **0.30** | **0.02** | [0.068; 1.231] |  |  |  |  |  |  |
| Age | **-0.29** | **0.03** | [-1.192; -0.040] |  |  |  |  |  |  |
| Sex | 0.03 | 0.76 | [-11.453; 15.447] |  |  |  |  |  |  |
| School attainment | -0.16 | 0.23 | [-3.144; 0.794] |  |  |  |  |  |  |
| BMI pre-surgery | 0.10 | 0.39 | [-0.594; 1.468] |  |  |  |  |  |  |
| BES | -0.24 | 0.07 | [-1.206; 0.059] |  |  |  |  |  |  |
| Bold values indicate significant variable. %EWL II, percentage of weight loss at the first follow up; SPM-E, Raven’s Standard Progressive Matrices age corrected; WCST, Winsconsin Card Sorting Test total score; BMI, Body Mass Index; BES, Binge Eating Scale. | | | | | | | | | |
